# Polymorphic regulation of mitochondrial fission and fusion modifies phenotypes of microglia in neuroinflammation

**DOI:** 10.1038/s41598-017-05232-0

**Published:** 2017-07-10

**Authors:** Mitsuhiko Katoh, Bao Wu, Huy Bang Nguyen, Truc Quynh Thai, Ryo Yamasaki, Haiyan Lu, Anna M. Rietsch, Musab M. Zorlu, Youichi Shinozaki, Yurika Saitoh, Sei Saitoh, Takashi Sakoh, Kazuhiro Ikenaka, Schuichi Koizumi, Richard M. Ransohoff, Nobuhiko Ohno

**Affiliations:** 1 Departments of Anatomy and Molecular Histology, Interdisciplinary Graduate School of Medicine and Engineering, University of Yamanashi, Chuo, Yamanashi, 409-3898 Japan; 2 Department of Histology and Embryology, Medical College of Chifeng University, Inner Mongolia, 024000 China; 3 Neuroinflammation Research Center, Lerner Research Institute, Cleveland Clinic, Cleveland, OH 44195 USA; 4 Neuropharmacology, Interdisciplinary Graduate School of Medicine and Engineering, University of Yamanashi, Chuo, Yamanashi, 409-3898 Japan; 5 Division of Neurobiology and Bioinformatics, National Institute for Physiological Sciences, Okazaki, Aichi 444-8787 Japan

## Abstract

Microglia are the resident macrophages of the central nervous system and play complex roles in the milieu of diseases including the primary diseases of myelin. Although mitochondria are critical for cellular functions and survival in the nervous system, alterations in and the roles of mitochondrial dynamics and associated signaling in microglia are still poorly understood. In the present study, by combining immunohistochemistry and 3D ultrastructural analyses, we show that mitochondrial fission/fusion in reactive microglia is differentially regulated from that in monocyte-derived macrophages and the ramified microglia of normal white matter in myelin disease models. Mouse cerebral microglia *in vitro* demonstrated that stimulation of TLR4 with lipopolysaccharide, widely used to examine microglial reactions, caused the activation of the mitochondrial fission protein, dynamin-related protein 1 (Drp1) and enhanced production of reactive oxygen species (ROS). The increase in the ROS level activated 5′ adenosine monophosphate-activated protein kinase (AMPK), and facilitated elongation of mitochondria along the microtubule tracks. These results suggest that the polymorphic regulation of mitochondrial fission and fusion in reactive microglia is mediated by distinct signaling under inflammatory conditions, and modulates microglial phenotypes through the production of ROS.

## Introduction

Microglia are specialized macrophages that reside in the central nervous system (CNS) and have distinct origins, morphologies and functions from other glial cells in the nervous system and macrophages in other organs^1, 2^. The morphological reactions of microglia are prominent in neurological disorders including the primary diseases of myelin. Furthermore, the morphology and gene expression of reactive microglia can be distinct from those of macrophages derived from infiltrating monocytes in neuroinflammation^3–5^. Microglial reactions are triggered by diverse stimuli and, most importantly, by neuronal injury or altered activity as well as exposure to plasma proteins after blood-brain barrier dysfunctions^6^. The activation of microglia has been classified into two phenotypes, pro-inflammatory (M1) and anti-inflammatory (M2), by analogies to peripheral macrophages; however, this classification is no longer regarded as accurate or useful^4^. Although dynamic changes in microglial functions may contribute to the phenotypes of various neurological disorders^7^, the regulatory mechanisms responsible for and associated changes in organelles underlying the complexity of microglial reactions are only beginning to be elucidated.

Mitochondria are critical for cellular metabolism and death, and their functions are controlled by their dynamics including fission/fusion^8–10^. Mitochondrial fission/fusion are regulated by molecules such as Dynamin-related protein 1 (Drp1) and Mitofusin (Mfn) 1/2^11–13^ and are mediated by other cellular components such as the cytoskeleton^14, 15^. Mitochondrial fission/fusion are also associated with the generation of reactive oxygen species (ROS). The role of ROS signaling has been demonstrated in a bewildering number of cellular events including damage to cell components. The production of ROS is also essential for host defenses in the periphery^16–18^. Therefore, mitochondrial fission/fusion and associated signaling involving ROS need to be investigated in microglia, due to their unique roles in brain physiology.

In the present study, the mitochondrial morphology of reactive microglia *in vivo* was examined and compared with those of monocyte-derived macrophages and microglia remote from tissue pathology, in myelin disease models. In order to model temporal alterations in mitochondrial fission/fusion in reactive microglia, we used primary microglial cultures and stimulation of Toll-like receptor 4 (TLR4) with lipopolysaccharide (LPS), which were widely used to study inflammatory reactions of microglia leading to injuries of neurons and oligodendrocytes^6, 19, 20^. The results suggest that microglial reactions induce dynamic mitochondrial fission/fusion which are associated with distinct signaling pathways. Alteration in mitochondrial dynamics may contribute to the differential functions of reactive microglia in neurological diseases.

## Results

### Mitochondrial dynamics of reactive microglia in demyelinating disease models *in vivo*

**Figure 1:**
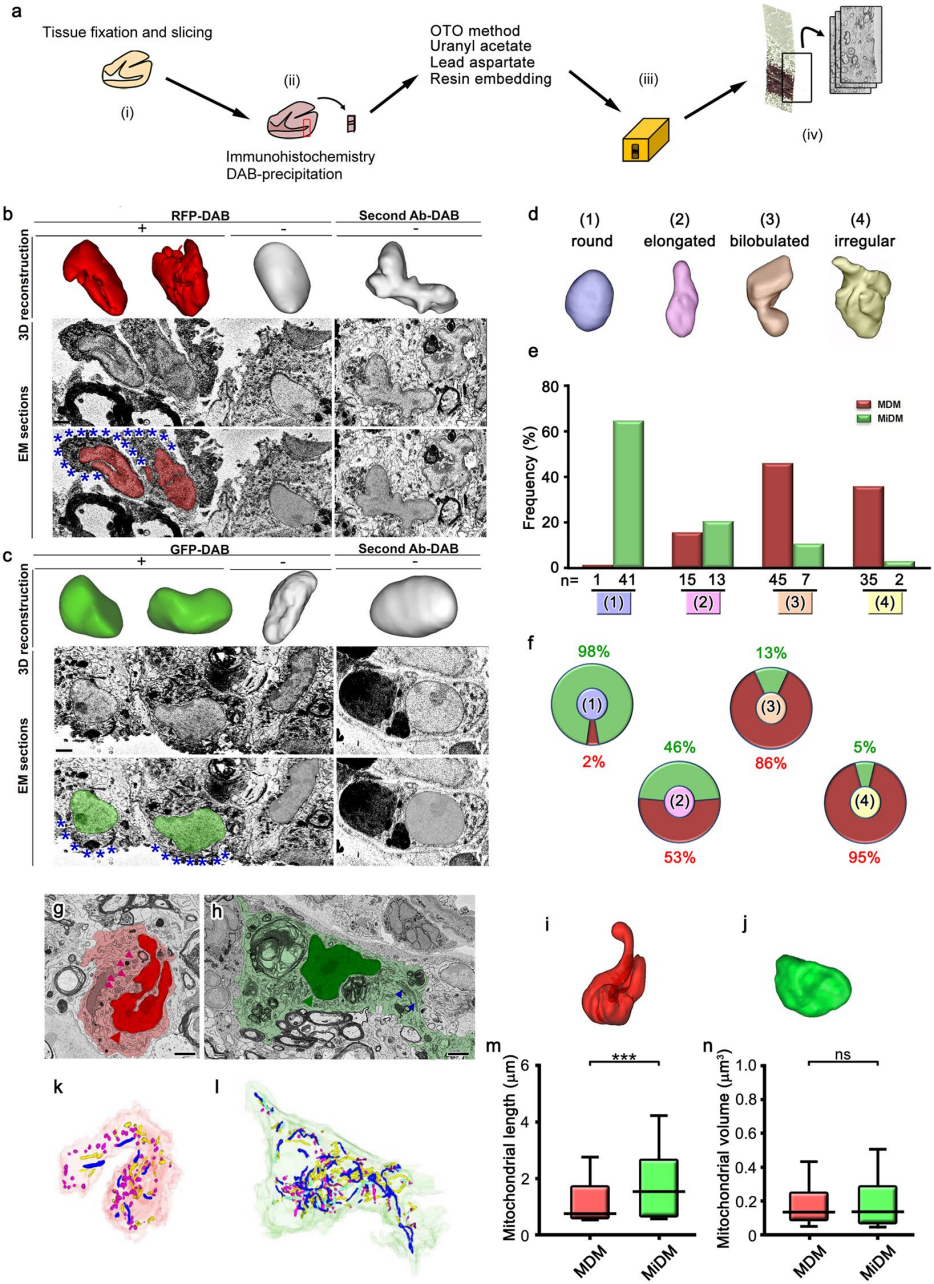
Longer mitochondria and round nuclei of microglia-derived macrophages in spinal cord tissues at the onset of experimental autoimmune encephalomyelitis. (**a**) After fixation and slicing (i), brain tissues were immunostained with DAB (ii), and embedded in resins (**a**, iii). Serial images were acquired for 3D reconstruction (**a**, iv). Reconstruction of nuclei (upper panels) and original immunoelectron microscopic images (middle, lower panels) of RFP- (**b**, red) and GFP- (**c**, green) positive cells in the spinal cord of a *Ccr2* ^*rfp/*+^::*Cx3cr1* ^*gfp/*+^ mouse with DAB deposits (**b,c**, asterisks). The nuclei of DAB negative cells (center) and immunocontrols (right) are also shown. The grading (**d**) and counting (**e**,**f**) of nuclear shapes in the RFP-positive monocyte-derived macrophages (MDM) and GFP-positive microglia-derived macrophages (MiDM). Single images (**g,h**) and nuclear (**i,j**) and mitochondrial (**k,l**) 3D reconstructions of MDM (**g,k,i**, red) and MiDM (**h,j,l**, green) are shown. Mitochondria are indicated (**g**,**h**, arrowheads). Long and short mitochondria are colored in blue and magenta, respectively (**k,l**). The mitochondrial length (**m**) and volume (**n**) of MiDM and MDM are compared. ns: not significant. ***p < 0.001 in the *U*-test. N = 648 or 786 for mitochondria in MDM or MiDM. Bars: 2 μm.

Using *Ccr2* ^*rfp/*+^::*Cx3cr1* ^*gfp/*+^ mice^21, 22^, which tissue-resident microglia-derived macrophages (MiDM) and infiltrating monocyte-derived macrophages (MDM) were labeled with GFP and RFP, respectively, the morphological characteristics of MiDM and MDM in the spinal cord at the onset of experimental autoimmune encephalomyelitis (EAE) were examined by immunostaining for RFP or GFP and observations with SBF-SEM (Fig. 1a). DAB-precipitation derived from GFP (MiDM) or RFP (MDM) immunostaining near the tissue surface facilitated the observation of organelles within the two populations of DAB-positive cells (Fig. 1b,c). In these analyses, the nuclei of GFP-positive MiDM (Fig. 1c) appeared to be more spherical compared with RFP-positive MDM (Fig. 1b). When nuclei were classified into 4 categories, 98% of cells with round nuclei were GFP+MiDM, while 95% of cells with irregular nuclei were RFP+MDM (Fig. 1d–f). MDM with complex nuclei had rounder and shorter mitochondria (Fig. 1g,k,i), whereas MiDM with round nuclei had longer mitochondria (Fig. 1h,l,j). Mitochondrial lengths were longer in MiDM than in MDM (Fig. 1m), while no significant differences were observed in mitochondrial volumes (Fig. 1n). These results suggest that MiDM have longer and thinner mitochondria and spherical nuclei than MDM in spinal cord tissues at the onset of EAE.

**Figure 2:**
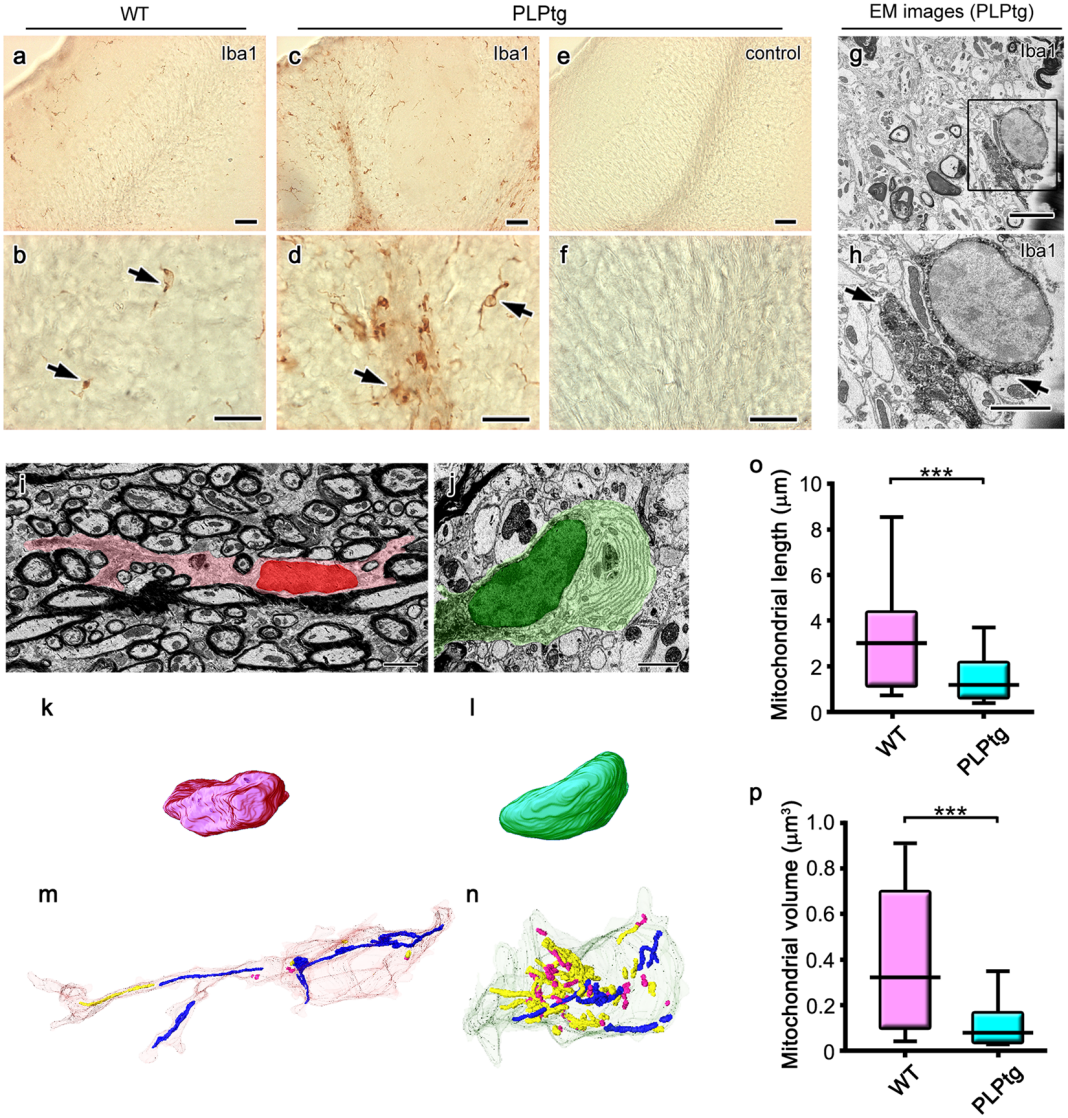
Shorter and smaller mitochondria predominate in amoeboidal microglia in demyelinated cerebellar white matter obtained from mice overexpressing proteolipid protein (PLP). Light micrographs of Iba1 immunostaining in the cerebella of 5-month-old wild-type (WT, **a,b**) and PLP-overexpressing (PLPtg, **c–f**) mice. Immunocontrol sections are also shown (**e,f**). In immunoelectron micrographs (EM) with DAB deposits for Iba1 (**g,h**, arrows), the area indicated with a rectangle (**g**) is magnified (**h**). Single electron micrographs (**i,j**) and 3D reconstructions of nuclei (**k,l**) and mitochondria (**m,n**) of Iba1-positive cells in the cerebellar white matter of WT (**i,k,m**) and PLPtg (**j,l,n**) mice are shown and mitochondrial length (**o**) and volume (**p**) were statistically compared. Long and short mitochondria are colored in blue and magenta, respectively (**m**,**n**). ***p < 0.001 in the *U*-test. N = 68 or 506 for mitochondria in WT or PLPtg microglia. Bars: 50 μm (**a,c,e**), 20 μm (**b,d,f**), 2.5 μm (**g–j**).

The differential fission/fusion of mitochondria may be related to the activation of macrophage lineage cells^18^. In order to address this possibility *in vivo*, mitochondrial morphologies were examined in a mouse model of Pelizaeus-Merzbacher disease (PMD), a congenital myelin disorder linked to the proteolipid protein (PLP) gene^23, 24^. In the mouse model expressing extra copies of PLP, progressive myelin loss and an increased number of macrophages were observed in the CNS, with most macrophages (>90%) being derived from resident microglia^25, 26^. Cells with DAB-deposits of ionized calcium-binding adapter molecule 1 (Iba1), a member of EF hand proteins which is expressed in the microglia of CNS as well as peripheral macrophages, were observed near the surface of the tissues (Figs 1a, 2a–h). In myelinated white matter from wild-type mice, Iba1-positive microglia had round-oval nuclei and extended thin processes containing long mitochondria (Fig. 2i,k,m). In contrast, in the demyelinated white matter of mutant mice, Iba1-positive amoeboid microglia with round-oval nuclei had greater numbers of small and short mitochondria than the ramified microglia in wild-type mice (Fig. 2j,l,n). The median length and volume of mitochondria was 2–3-fold longer and larger in ramified microglia compared with the amoeboid microglia (Fig. 2o,p). These results suggest that reactive microglia are characterized more by a constant nuclear morphology and mitochondrial fragmentation than ramified microglia *in vivo*.

### Transient fragmentation and subsequent elongation of mitochondria in reactive microglia stimulated with LPS *in vitro*

**Figure 3:**
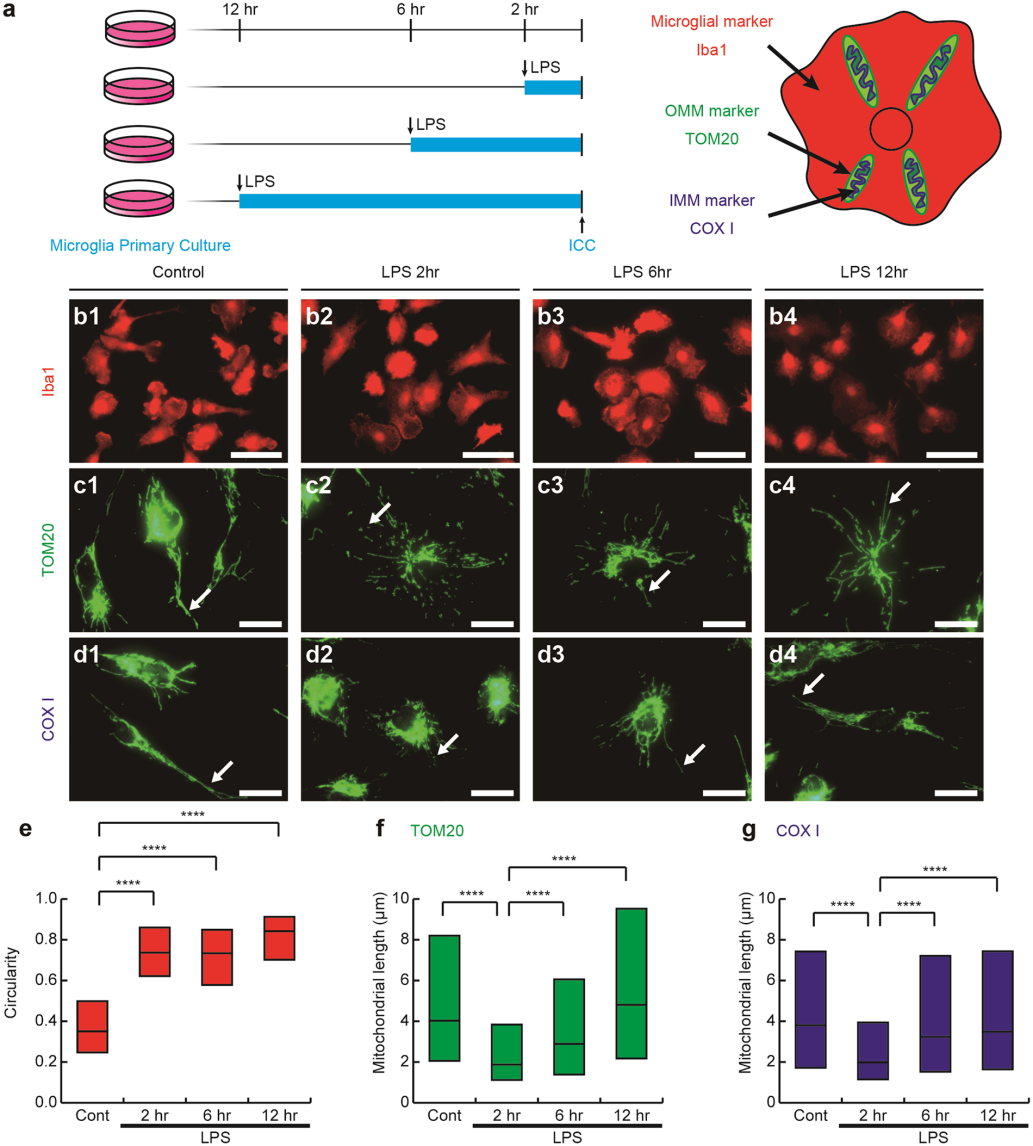
A lipopolysaccharide (LPS) stimulation induces fragmentation and the subsequent elongation of mitochondria in primary microglial cultures. (**a**) Primary cultures of microglia were incubated with or without LPS for 2, 6 and 12 hr and then immunocytochemically stained (ICC) for markers of microglia (Iba1), the outer mitochondrial membrane (OMM and TOM20), and inner mitochondrial membrane (IMM and COX I). Representative images of immunofluorescence for Iba1 (b1-4), TOM20 (c1-4), and COX I (d1-4), the circularity (proportional to cellular area/perimeter^2^) (**e**), and mitochondrial length measured as the length of TOM20- (**c**, arrows, **f**) and COX I-positive (**d**, arrows, **g**) profiles are shown. Cont: unstimulated cultures. ****p < 0.0001 in the *U*-test. N (Cont, 2 hr, 6 hr, 12 hr) = (130, 120, 128, 113) cells in (**e**), (2538, 3626, 3162, 2259) mitochondria in (**f**), and (1135, 1704, 1280, 1348) mitochondria in (**g**). Medians (bars) and quartile ranges (boxes) are shown (**e–g**). Bars: 50 μm (**b**), 20 μm (**c,d**).

**Figure 4:**
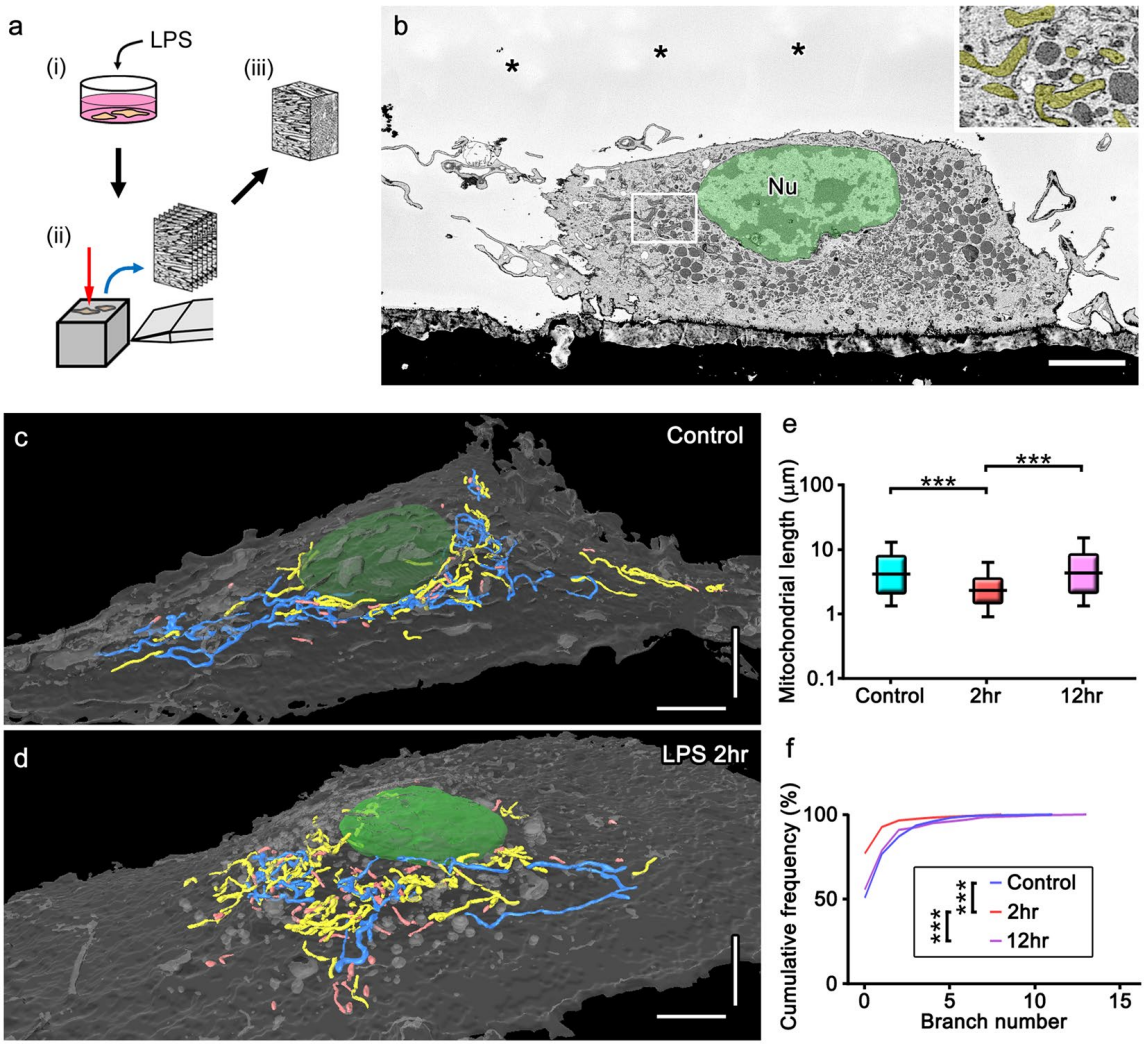
Electron microscopic 3D reconstructions showing mitochondrial fragmentation and subsequent elongation with network restoration in cultured microglia after a stimulation with lipopolysaccharide (LPS). Cultured microglia with or without the LPS stimulation (**a**, i) were fixed and embedded in resins to image with SBF-SEM (**a**, ii) and mitochondria were reconstructed from the serial images (**a**, iii). Mitochondria with typical cristae (**b**, yellow) were reconstructed and colored (**c,d**) in cultured microglia not stimulated with LPS (Control, **c**) and 2 hr after the LPS stimulation (**d**, LPS 2hr). Measurements of length (**e**) and branch number (**f**) are shown. Long and short mitochondria are colored in blue and pink, respectively (**c,d**). ***p < 0.001 in the *U*-test. Bars: 5 μm.

**Figure 5:**
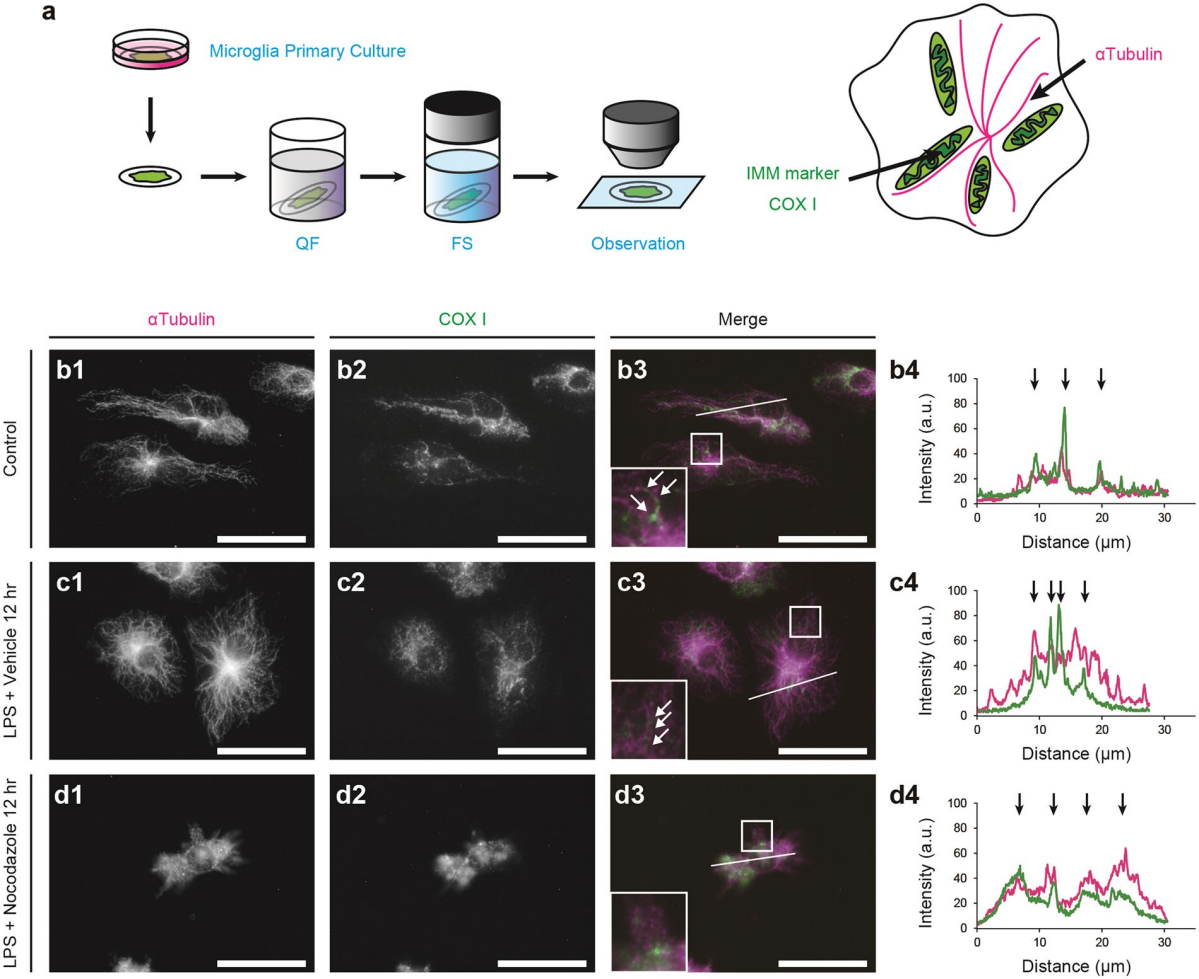
Mitochondrial elongation along microtubules following the stimulation with lipopolysaccharide (LPS). An experimental scheme shows that primary microglial cultures were quickly frozen (QF), freeze-substituted (FS), and immunostained for α-tubulin and the inner mitochondrial membrane (IMM) marker, COX I (**a**). Double immunostaining for mitochondria (COX I) and microtubules (α-tubulin) in microglia without the LPS stimulation (**b**, arrows) or 12 hr after the LPS stimulation (**c**, arrows). Areas indicated with rectangles (b3, c3, d3) are shown in the insets. Fluorescence intensity was measured along the lines (b3, c3, d3), and the arrows show the strong fluorescence intensities of α-tubulin (magenta) and COX I (green) at the same locations (b4, c4). Bars: 30 μm.

In order to elucidate mitochondria alterations and associated signaling pathways during microglial reactions, primary cultures of microglia were utilized and subjected to 1 μg/mL LPS, which cause inflammatory reactions of microglia by signaling to TLR4 (Fig. 3a)^6, 19, 20, 27^. Immunostaining for Iba1 showed that the circularity of the cytoplasm (proportional to the cellular area/perimeter^2^) was greater in cultures 2, 6 and 12 hr after the stimulation than in unstimulated control cultures (Fig. 3b,e), indicating that the LPS stimulation induces the amoeboid morphology. Immunostaining was used to elucidate the mitochondrial outer membranes (TOM20; Fig. 3c, VDAC1; data not shown) and inner membranes (COX I; Fig. 3d, COX Va; data not shown). Mitochondrial length was shortened 2 hr after the stimulation (Fig. 3f,g). Mitochondria were longer 6 and 12 hr after the stimulation than 2 hr after the stimulation (Fig. 3f,g). SBF-SEM observation and the reconstruction of mitochondria identified by the typical cristae morphology (Fig. 4a,b) showed that the lengths and numbers of branches were significantly shorter and fewer, respectively, 2 hr after the LPS stimulation (Fig. 4c–f), and returned to control levels 12 hr after the stimulation (Fig. 4e,f). We speculated that the elongation of mitochondria was dependent on microtubules, because double immunostaining for α-tubulin and COX I showed that elongated mitochondria co-localized with microtubules (Fig. 5a–c). This hypothesis was confirmed by nocodazole, an inhibitor of microtubule polymerization, impairing the elongation of mitochondria (Fig. 5d). These results demonstrate that the activation of microglia by LPS induced transient and reversible shortening and the subsequent elongation and network restoration of mitochondria *in vitro*.

### Drp1 activation and mitochondrial ROS production mediating LPS-induced mitochondrial fragmentation

**Figure 6:**
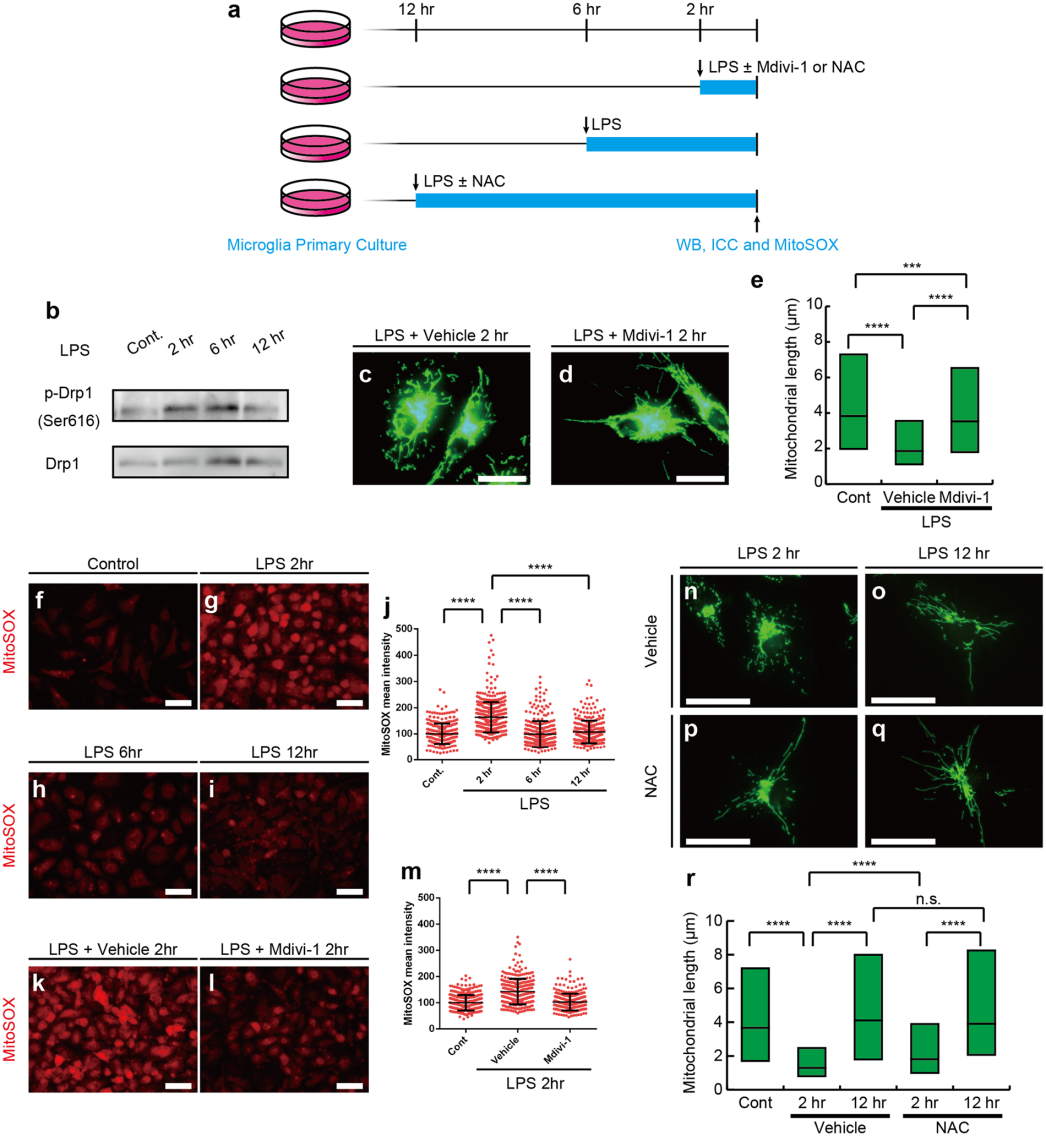
Lipopolysaccharide (LPS)-induced mitochondrial fragmentation in microglia is controlled by Drp1 signaling and evokes mitochondrial reactive oxygen species (ROS) generation. Primary microglial cultures were incubated without LPS (Cont) or with LPS for 2 (2hr), 6 (6hr) and 12 hr (12hr), and subjected to Western blotting analyses for total Drp1 and Drp1 activated by the phosphorylation at Ser616 (p-Drp1) or MitoSOX analyses to detect mitochondrial ROS (**a**). Primary microglial cultures were stimulated by LPS for 2 hr with or without an inhibitor for Drp1 (Mdivi-1) or N-acetyl cysteine (NAC) and subjected to immunostaining or MitoSOX analyses (**a**). Western blotting of phosphorylated Drp1 and total Drp1 following LPS stimulation. (**b**). Representative images (**c,d**) and measured lengths (**e**) of TOM20-immunopositive mitochondrial profiles are shown. N = 2672 (Cont), 5040 (Vehicle), and 2820 (Mdivi-1) mitochondria in (**e**). MitoSOX images of primary microglial cultures without the LPS stimulation (Cont, **f**) or with the LPS stimulation for 2 (2hr, **g**), 6 (6hr, **h**) or 12 (12hr, **i**) hr are shown and quantified (**j**). N = 287 (control), 444 (LPS 2hr), 318 (LPS 6hr) and 309 (LPS 12hr) cells. Each dot represents each cell. Representative images (**k,l**) and the mean fluorescence intensity (**m**) of MitoSOX in microglial cultures 2 hr after LPS stimulation with (**l**) or without (**k**) Mdivi-1 treatment. N = 336 (Cont), 306 (Vehicle) and N = 297 (Mdivi-1) cells. Representative images (**n–p**) and measured mitochondrial length (**r**) after 2hr (**n,p**) or 12hr (**o,q**) LPS stimulation of Toll-like receptor 4 (TLR4) with (**p,q**) or without (**n,o**) NAC treatment. N = 173 (Cont), 570 (2hr-Vehicle), 411 (12hr-Vehicle), 572 (2hr-NAC), and 364 (12hr-NAC) mitochondria in (r). ***p < 0.001, ****p < 0.0001 and n.s.: not significant in the *U*-test. Medians (**e,j,m,p**, bars) with quartile ranges (**e,p**, boxes; **j**,**m**, whiskers) are shown. Bars: 20 μm (**c,d**, **n-q**), 50 μm (**f–i**, **k,l**).

Since the activation of Drp1 controls mitochondrial fission^28^, we examined Ser616 phosphorylation, which activates Drp1 after the LPS stimulation (Fig. 6a). Western blotting analyses revealed that the phosphorylation of Ser616 increased 2–6 hr after the stimulation, and returned to control levels 12 hr after the stimulation (Fig. 6b). The treatment with 50 μM Mdivi-1, a Drp1 inhibitor (Fig. 6a), diminished mitochondrial shortening (Fig. 6c–e)^29^. Drp1 activation and mitochondrial fission have been shown to promote the generation of ROS^30^. In order to examine alterations in and the roles of ROS production after the LPS stimulation, mitochondrial ROS (mtROS) was measured with MitoSOX, a mitochondrial superoxide indicator (Fig. 6a). The fluorescence intensity of MitoSOX increased 2hr after the LPS stimulation (Fig. 6f,g,j) and returned to the basal levels 6–12 hr after the stimulation (Fig. 6h,i,j). The treatment with Mdivi-1 abolished the increase in MitoSOX fluorescence intensity observed 2hr after the LPS stimulation (Fig. 6a,k–m). The generated ROS appeared to contribute mitochondrial fragmentation 2hr after the LPS stimulation, since treatment with 1 mM N-acetyl cysteine (NAC), a ROS scavenger, diminished the mitochondrial fragmentation (Fig. 6n–r). Mitochondrial length after 12hr treatment with NAC was comparable to those with 12hr treatment with LPS and controls without LPS stimulation, but longer than that with 2hr treatment with LPS and NAC (Fig. 6n–r). Since IL-10 is a pivotal regulator in progression of EAE and the production of IL-10 is increased by LPS stimulation and promoted by post-translational modification of microtubules^31–33^, we examined secretion levels of IL-10 by enzyme-linked immunosorbent assay (ELISA). Although LPS stimulation increased production of IL-10, treatment with Mdivi-1 did not significantly affect the IL-10 production (Supplementary Fig. 1). These results suggest that mitochondrial fission via the activation of Drp1 is induced by the TLR4 stimulation and increases the mitochondrial production of ROS which contributes to mitochondrial fission.

### Mitochondrial elongation mediated by production of ROS and activation of AMPK

**Figure 7:**
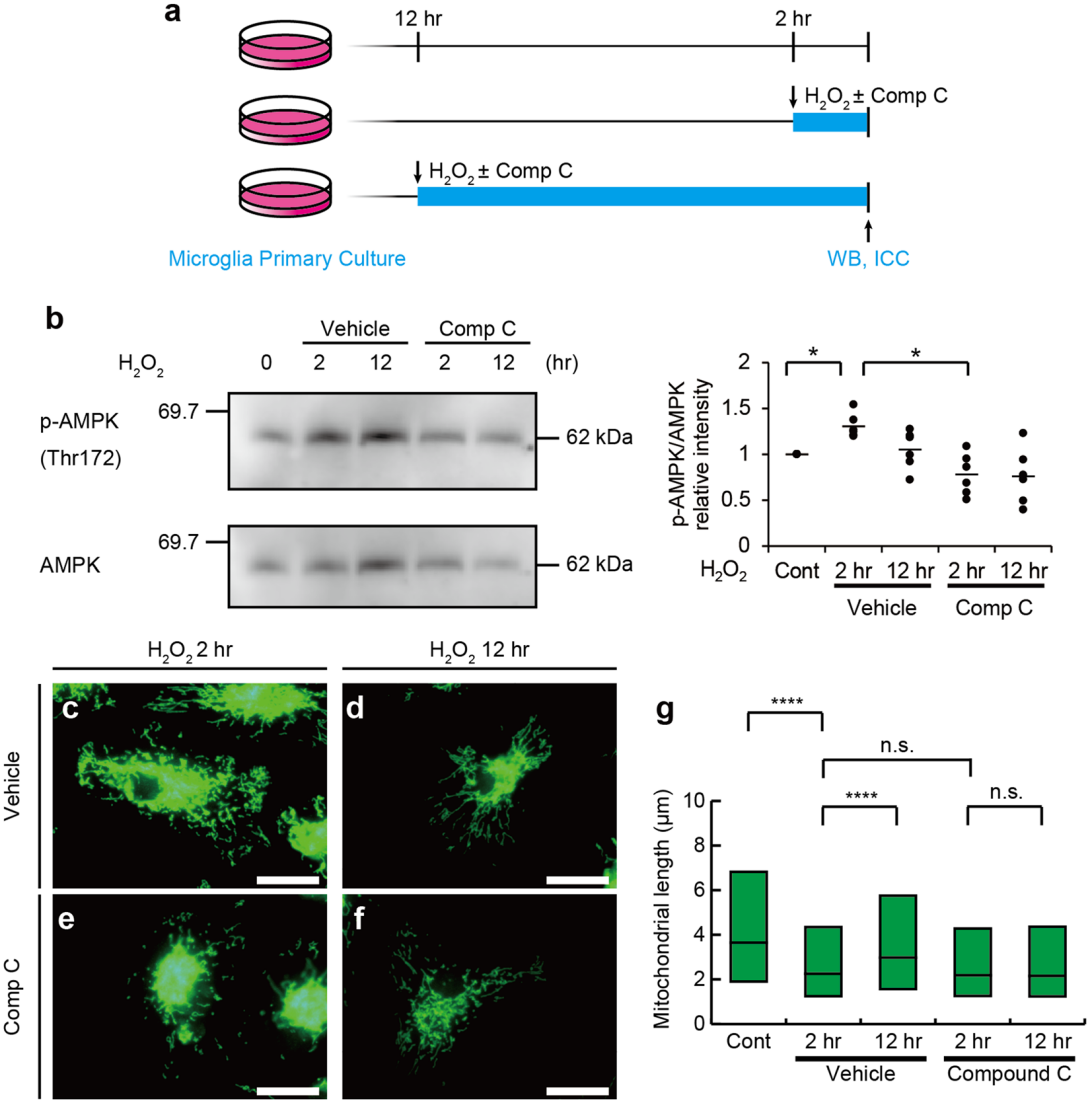
ROS activates 5′ adenosine monophosphate-activated protein kinase (AMPK) to facilitate mitochondrial elongation. (**a**) Primary microglial cultures were treated for 2 or 12 hr with or without hydrogen peroxide (H_2_O_2_) and the selective AMPK inhibitor, Compound C (Comp C), and then subjected to Western blotting (WB) and immunostaining (ICC). Western blotting of AMPK phosphorylated at Thr172 (p-AMPK) with treatments of H_2_O_2_ and Comp C (**b**, left), and quantification of the Western blotting analyses (**b**, right) are shown. *p < 0.05 in the t-test (N = 6). Each dot represents each replicate and means (bars) are shown. Representative images (**c–f**) and the measured lengths (**g**) of TOM20-immunopositive mitochondrial profiles with treatments of H_2_O_2_ and Comp C. N = 1975 (control), 4536 (H_2_O_2_ 2 hr), 3156 (H_2_O_2_ 12 hr), 3486 (H_2_O_2_ 2 hr + Comp C), and 2966 (H_2_O_2_ 12hr + Comp C) for mitochondria. n.s.: not significant and ****p < 0.0001 in the *U*-test. Medians (**g**, bars) with quartile ranges (**g**, boxes) are shown. Bars: 20 μm.

Increase in superoxide activate 5′ adenosine monophosphate-activated protein kinase (AMPK) and facilitate mitochondrial biogenesis^34–36^. When microglia were treated with 1 μM hydrogen peroxide (H_2_O_2_) for 2 or 12 hr (Fig. 7a), the phosphorylation of AMPK at Thr172, which activates AMPK, was induced (Fig. 7b). Incubation with H_2_O_2_ in the presence of 5 μM Compound C (Dorsomorphin), a selective AMPK inhibitor (Fig. 7a)^37^, decreased AMPK phosphorylation (Fig. 7b). Mitochondrial length was shorter 2 hr after the treatment with hydrogen peroxide, and returned to basal levels 12 hr after the stimulation (Fig. 7c,d,g). Mitochondrial elongation after the hydrogen peroxide treatment was inhibited by Compound C (Fig. 7e,f,g).

**Figure 8:**
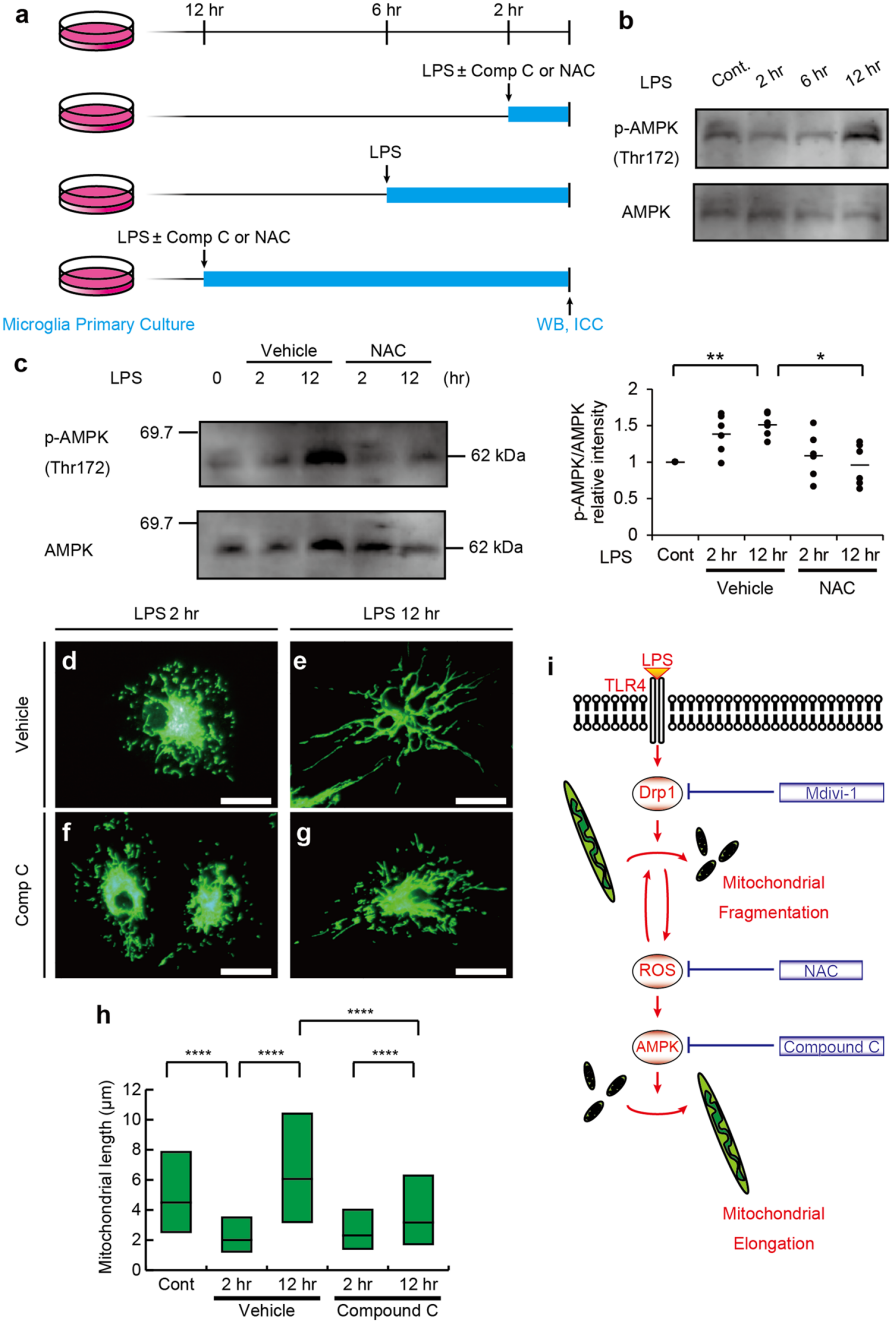
Mitochondrial elongation after a 12 hr stimulation with lipopolysaccharide (LPS) is mediated by reactive oxygen species (ROS) and activated AMP-activated protein kinase (AMPK). Primary microglial cultures were untreated (Cont) or treated with LPS for 2 (2hr) or 12 (12hr) hr with or without drugs including a ROS scavenger (N-acetyl cysteine; NAC) and Compound C (Comp C), and then subjected to Western blotting and immunostaining for TOM20 (**a**). Western blotting of AMPK phosphorylated at Thr172 (p-AMPK) and total AMPK following LPS stimulation (**b**). Western blotting of p-AMPK with or without NAC treatment (**c**, left) and quantification of the western blotting analyses (**c**, right) are shown. *p < 0.05, **p < 0.01 in t-test (N = 6). Each dot represents each replicate and means (bars) are shown. Representative images (**d–g**) and the measured lengths (**h**) of TOM20-immunopositive mitochondrial profiles with or without treatments of NAC (**h**). The graphical summary of the model is shown (**i**). N = 1818 (control), 3489 (LPS 2hr), 2238 (LPS 12hr), (LPS 2hr + Comp C) and 3654 (LPS 12hr + Comp C) mitochondria in (**h**). ****p < 0.0001 in the *U*-test. Medians (**h**, bars) with quartile ranges (**h**, boxes) are shown. Bars: 20 μm.

In order to confirm the involvement of ROS and AMPK in mitochondrial fission/fusion after the LPS stimulation, microglial cultures were stimulated with LPS in the presence or absence of NAC, and Compound C (Fig. 8a). Western blotting analysis showed that stimulation by LPS for 12 hr induced phosphorylation of AMPK at Thr172 (Fig. 8b). LPS-induced phosphorylation of AMPK at Thr172 was decreased by the treatment with NAC (Fig. 8c). Compound C suppressed mitochondrial elongation observed 2–12 hr after the LPS stimulation (Fig. 8d–h), but did not significantly affect production of IL-10 (Supplementary Fig. 1). These results suggest that mitochondrial elongation in microglia after the LPS stimulation is mediated by the production of ROS and activation of AMPK (Fig. 8i).

## Discussion

In the present study using SBF-SEM, the mitochondrial morphology of reactive microglia was compared with those of monocyte-derived macrophages and microglia from unaffected brain regions, under demyelinating conditions *in vivo*. Temporal changes in mitochondrial morphology and associated signaling were also evaluated in primary microglial cultures after the LPS stimulation, using immunocytochemistry and SBF-SEM. Morphological analyses *in vivo* revealed that mitochondria were longer in MiDM than in MDM at the onset of EAE. On the other hand, the mitochondria of amoeboid microglia in the demyelinated white matter of PLP-overexpressing mice were shorter than those of ramified microglia in the myelinated white matter of wild-type mice. The results of *in vitro* analyses demonstrated (1) transient mitochondrial shortening after the LPS stimulation via pathways involving Drp1 and ROS, and (2) subsequent elongation along microtubule tracks via pathways involving AMPK (Fig. 8i). Since mitochondrial shortening enhanced the production of ROS in microglia, these results suggest that endogenous signaling pathways in reactive microglia lead to influence mitochondrial dynamics and modulate microglial metabolism and ROS production in neurological disorders.

SBF-SEM revealed the distinct morphology of macrophage populations in detailed 3D ultrastructural analyses of demyelinated mouse CNS tissues and primary microglial cultures. The biphasic alterations in mitochondrial length observed at the ultrastructural level *in vitro* were consistent with immunohistochemical staining for the markers of the inner and outer mitochondrial membranes, indicating that light and 3D electron microscopic methods are mutually complementary^38, 39^. In addition, SBF-SEM observations along with pre-embedding immunohistochemistry for extrinsic and intrinsic markers clarified nuclear and mitochondrial morphologies in different MiDM and MDM populations *in vivo*. The penetration of the immunoreaction product was limited near the surface, but sufficient to identify immunostained cells and their organelles. These approaches may facilitate the identification of ultrastructural features in specific cell populations and tissue compartments under physiological and pathological circumstances.

The 3D morphological *in vivo* analyses suggested the differential nuclear and mitochondrial morphologies of macrophages in neuroinflammatory and demyelinating diseases. The complexity of nuclear shapes may be relevant to the differential properties of nuclear cytoskeletal elements, including the effects of migratory behaviors, such as extravasation from blood vessels^40, 41^. Mitochondrial morphology is closely associated with cellular metabolism and functions in neurons and glia^42, 43^. In peripheral immune cells, metabolic pathways are associated with various immunological functions^18, 44^. For example, an inflammatory stimulation of macrophages such as that with LPS enhances glycolytic metabolism, inhibits oxidative phosphorylation and increases ROS production from mitochondria^44^. The generation of ROS has been correlated with mitochondrial fragmentation and the secretion of inflammatory cytokines in cell lines^45, 46^. MDM have been reported to express higher levels of inflammatory cytokines than MiDM at the onset and peak of EAE^5^. Differences in organelle morphology between MiDM and MDM support the concept that the behaviors and functions of these two populations of macrophages are distinct *in vivo* and differentially associated with pathogenesis and potentially with tissue repair in this model of inflammatory demyelination.

Alterations of the mitochondrial dynamics may be related to the differences of microglial phenotypes in the demyelinating disease models. Amoeboid microglia were observed in PLP-overexpressing mice although MiDM in EAE onset bore processes^5^. Inflammation-associated genes were upregulated in MDM rather than MiDM in EAE^5^, whereas inflammatory reactions including phospholipase D4 (PLD4), involved in M1 macrophage polarization, was induced in reactive microglia of PLP-tg mice^47, 48^. However, the limitation of this study is the different brain regions analyzed in different models in order to observe microglial reactions in demyelinated white matter. Further studies on variances of brain regions as well as genetic backgrounds are necessary in order to address if mitochondrial dynamics of reactive microglia is altered in the different demyelination models.

The *in vitro* analyses demonstrated time-dependent alterations of mitochondrial dynamics and their roles in ROS production in microglia following the inflammatory stimulation of TLR4 with LPS. TLR4 has been implicated in pathophysiology of EAE^49, 50^. Although involvement of TLR4 is not well understood in the demyelination by PLP overexpression, previous reports suggested that PLD4, involved in M1 macrophage polarization, was induced both in LPS-stimulated primary microglial cultures and in the demyelination model by PLP overexpression^47, 48^. Thus, it is possible that microglial reactions in PLP-tg and after LPS stimulation at least partly share common signaling pathways. In addition, LPS stimulation of TLR4 has been widely used to investigate inflammatory microglial reactions^19, 20, 27^. Therefore, the mitochondrial changes and their roles in microglial reactions observed in this study are potentially observed in the other neuroinflammatory circumstances. However, the *in vivo* environment is difficult to be reproduced *in vitro* ^4^, and the phenotypes of reactive microglia in EAE and PLP-tg are unlikely to be uniform. Further studies with different stimulations as well as approaches using *in vivo* observation and manipulation of mitochondrial dynamics are needed in order to understand the mechanisms and roles of mitochondrial changes in microglia more deeply *in vivo*.

Morphological changes in mitochondria *in vitro* are controlled via signaling involving Drp1 in the early phase and ROS-AMPK in the later phase. Multiple mitogen-activated protein kinase (MAPK) pathways lie downstream of the LPS stimulation. It is tempting to speculate that the phosphorylation and activation of Drp1 by specific MAPK signaling pathways may mediate mitochondrial fragmentation, dependent on cellular types and/or metabolic states^51, 52^. Adding potential complexity, other signaling events may induce mitochondrial fragmentation via the phosphorylation and degradation of Mfn2^53, 54^, rather than the activation of Drp1. The production of ROS has been correlated with mitochondrial fragmentation induced by the activation of Drp1^45, 55, 56^. The mitochondrial fragmentation in LPS-stimulated microglia was not due to apoptotic cell death or cell cycle propagation^57, 58^, because (1) cellular loss as well as apoptotic morphology was minimal after LPS stimulation and (2) mitochondrial fragmentation was observed in most cells (data not shown). ROS exert various effects; they induce cell damage or promote survival, depending on their concentration and distribution^59^. The results of the present study suggest that ROS enhanced mitochondrial fragmentation in the early phase, but activated AMPK, which led to mitochondrial elongation in the later phase. AMPK signaling is critical for mitochondrial biogenesis via the induction of peroxisome proliferator-activated receptor gamma coactivator 1-α^60^. Therefore, these results support the concept that mitochondrial fragmentation and elongation mediated by the dynamic activation of specific pathways contribute to microglial dysfunctions under the conditions of altered CNS homeostasis. Further investigations on the roles of mitochondrial dynamics particularly such as transcription and cytokine productions would be necessary to understand precise roles of mitochondrial dynamics in microglial reactions, since our results suggested that inhibition of mitochondrial fission did not significantly affect LPS-induced production of IL-10. However, our results support the possibility that the restoration of the form and function of microglial mitochondria provides a salient avenue for therapeutic manipulations ameliorating ROS-mediated tissue injuries.

## Materials and Methods

### Animals, EAE induction and clinical evaluations

All animal experiments were approved by the Institutional Animal Care and Use Committee of University of Yamanashi or Cleveland Clinic, and were performed in accordance with relevant guidelines and regulations. *Ccr2* ^rfp/+^::*Cx3cr1* ^gfp/+^ mice were generated by crossing *Ccr2* ^rfp/rfp^::C57BL/6 mice with *Cx3cr1* ^gfp/gfp^::C57BL/6 mice^22, 61^. PLPtg mice^23^ were obtained from Riken BRC (Tsukuba, Ibaraki, Japan) and maintained by crossing with DBF-1 mice for >5 generations. Male PLPtg mice and control littermates were used in experiments. DBF-1 and C57BL/6 mice were obtained from Japan SLC (Shizuoka, Japan) or the National Cancer Institute.

EAE was induced in *Ccr2* ^rfp/+^::*Cx3cr1* ^gfp/+^ mice using myelin-oligodendrocyte-glycoprotein peptide 35–55 (MOG), and all mice were weighed and graded daily for clinical stages, as previously reported^5^. The onset stage of EAE was defined as the day on which EAE signs appeared.

### Primary microglial culture

Primary microglial cultures were prepared as previously reported^62, 63^. Briefly, the cerebral cortices of newborn C57BL/6 mice were minced and cultured with Dulbecco’s Modified Eagle’s Medium (DMEM) containing 10% fetal bovine serum (FBS) in a 75 cm^2^ flask for approximately 10–14 days. Microglia were obtained by gently shaking the flask (approximately 100 rpm) for 10 min. The purity of microglia was assessed as >99% by immunostaining for markers (data not shown). Gathered microglia were cultured in DMEM supplemented with 10% FBS and subjected to experiments. LPS and Mdivi-1 were purchased from Sigma-Aldrich Japan (Tokyo, Japan). Compound C (Dorsomorphin) was purchased from Wako (Tokyo, Japan).

### Immunocytochemistry and mitochondrial length measurements in cultured microglia

In immunostaining for Iba1, TOM20, VDAC1, COX I and COX Va, microglia were fixed with 4% paraformaldehyde at 4 °C overnight, and then permeabilized in PBS with 0.5% Triton-X for 1hr. In immunostaining for α-tubulin, microglia were subjected to freeze-substitution (FS) followed by quick-freezing (QF), as previously reported^64^. Briefly, microglial cultures on slide glasses were dipped into isopentane-propane cryogen (−193 °C) and incubated in acetone with dry ice (−80 °C) overnight. The temperature of acetone was gradually increased by incubating the samples at −20 °C, 4 °C, and room temperature for 2 hr each. Samples were incubated with the primary antibody in PBS with 0.1% Triton-X and 5% BSA at 4 °C overnight. Cells were then washed with PBS and incubated with secondary antibodies (Alexa-conjugated) in PBS with 0.1% Triton-X and 5% BSA. The primary antibodies were Iba1 (Wako, 019–19741, 1:2000), TOM20 (Santa Cruz, sc-11415, 1:1000), VDAC1 (Abcam, ab14734, 1:900), COX I (Abcam, ab14705, 1:400), COX Va (Novus Biologicals, NBP1-32550, 1:300), and α-tubulin (Abcam, ab52866, 1:1500).

Mitochondrial lengths were measured with ImageJ following immunocytochemistry for mitochondrial markers. All countable mitochondria in cells were traced by free-hand lines and their lengths were measured. All immunocytochemical experiments for each mitochondrial marker were repeated three times with at least 50 cells in total being measured.

### Mitochondrial ROS measurements

ROS generated by mitochondria were measured using MitoSOX red as described previously^65^. Briefly, cells were incubated with 5 μM MitoSOX for 10 min and washed with medium at 37 °C. Cells were fixed with 4% paraformaldehyde at room temperature for 10 min. Samples were observed by fluorescent microscopy and mean MitoSOX intensity in the cytoplasm was measured using Image J as described previously^66^.

### Western blotting analysis

Microglia in the primary culture were lysed using Laemmli sample buffer (0.5 Tris-HCl [pH 6.8] 12.5 mL, Glycerol 10 mL, 10% SDS 20 mL, 2-mercaptoethanol 50 μL and BPB) with 1% phosphatase inhibitor. Samples were subjected to SDS-PAGE and Western blotting. The primary antibodies used were anti-phospho-Drp1 (Ser616) (Cell Signaling Technology), anti-DLP1 (BD, 611112), anti-phospho-AMPKα (Thr172) (Cell Signaling Technology) and anti-AMPKα (Cell Signaling Technology) and all primary antibodies were diluted 1:1000. The second antibodies used were goat anti-rabbit IgG (H+L), HRP conjugate (Thermo, #31460) and HRP horse anti-mouse IgG antibody (Peroxidase) (Vector Laboratories, PI-2000) and all secondary antibodies were diluted 1:3000. We detected signals using chemiluminescence system.

### Serial block-face scanning electron microscopy (SBF-SEM) observations with/without immunohistochemistry

Tissues were removed after mice have been perfusion-fixed using buffered 4% PFA with 1–2.5% glutaraldehyde. Cultured microglia were fixed by immersion in buffered 4% PFA with 0.5% glutaraldehyde. Tissues were immersed in the same fixatives overnight. Brain tissues for immunoelectron microscopy were sliced with a vibratome after fixation, and incubated with primary antibodies against GFP, RFP (Company), and Iba1 (Wako Chemicals). Immunoreaction products were visualized uing the avidin-biotin-horse radish peroxidase complex (ABC) method (Vector) and DAB substrate kit (Pierce, Rockford, IL, USA), and additionally fixed with 4% PFA with 1% glutaraldehyde. In immunostaining for anti-GFP or RFP antibodies, glutaraldehyde was omitted from the fixation step before immunostaining. Samples for SBF-SEM observations were post-fixed with osmium and stained *en bloc*, as described previously^67^. Following trimming, samples were imaged with Sigma VP or Merlin (Zeiss) equipped with 3View (Gatan Inc.). The serial images acquired were handled and processed for segmentation with Fiji/ImageJ.

### Mitochondrial measurements and nuclear grading in SBF-SEM images

Mitochondria in SBF-SEM images were segmented and reconstructed to 3D images using TrakEM2^68^ and Amira software. Their lengths, volumes and branch numbers were measured or counted from the segmented and tracked mitochondrial profiles in each image with TrakEM2. The characterization of nuclear shapes was conducted using SBF-SEM images, as described previously^5^. Nuclei were categorized as follows: (1) “Round”, having a round shape and smooth surface with a ratio of length/width ≤ 1.5 with or without small (<half width) indentations; (2) “Elongated”, having an elongated or oval shape with a length/width ratio >1.5 with or without small (<half width) indentations; (3) “Bilobulated”, having two connected lobes with a single intervening large (>half width) indentation; (4) “Irregular”, having complex shape with a corrugated surface, and potentially with multiple and variable sized indentations. Three blinded observers including a research student, research fellow, and neuroscientist scored nuclear morphologies from serial images obtained using SBF-SEM. Observers were trained on the same nuclear examples in each category and practiced using 20 nuclei comprising all shapes before scoring the nuclei. The Kappa test showed good pairwise agreement rates among observers (0.85–0.90) and data from the research fellow were used.

### ELISA of IL-10

IL-10 produced by primary microglial culture was measured by Mouse IL-10 ELISA MAX™ Deluxe (BioLegend, Inc.) following manufacture’s protocol. The absorbance at 570 nm of standards and samples were subtracted from the absorbance at 450 nm. Experiment was repeated at least 4 times.

### Statistical analysis

Quantifications in the EAE model were performed on 3 individual mice from 3 EAE inductions including 28–35 cells from two separate lesions from each mouse in the assay. Quantifications in PLPtg mice were performed on 3 individual mice from 3 littermates including 6–9 cells from at least 2 different sites of cerebellar white matter. In measurements of the mitochondrial lengths of cultured microglia, experiments were repeated at least 3 times and all data were shown in box graphs which lines were medians and boxes were first-to-third quartile ranges. MitoSOX experiments were performed at least 3 times and representative data were presented in plots. Statistical analyses were performed using Prism (GraphPad Software, La Jolla, CA). Mitochondrial lengths and volumes were measured with ImageJ software, and comparisons were made by the Mann-Whitney *U* test. Intensity of bands in Western blotting was measured by Image J, and each blot was normalized against control. Comparisons were made by two-tailed Student t tests with Bonferroni corrections. Graphs show medians (bars), quartile ranges (boxes), and 10–90% ranges (whiskers), unless specifically stated.

## Electronic supplementary material

Supplementary information
