# Supplementary material for: Polymorphic regulation of mitochondrial fission and fusion modifies phenotypes of microglia in neuroinflammation
Source: Sci Rep. 2017 Jul 10;7:4942. doi: 10.1038/s41598-017-05232-0 (PMC5503944; doi:10.1038/s41598-017-05232-0)
Supplement: Supplementary file 1 — Supplementary information [file 41598_2017_5232_MOESM1_ESM.pdf]

Polymorphic regulation of mitochondrial fission and fusion modifies phenotypes of microglia in neuroinflammation

Mitsuhiko Katoh, Bao Wu, Huy Bang Nguyen, Truc Quynh Thai, Ryo Yamasaki, Haiyan Lu, Anna M. Rietsch, Musab M. Zorlu, Youichi Shinozaki, Yurika Saitoh, Sei Saitoh, Takashi Sakoh, Kazuhiro Ikenaka, Schuichi Koizumi, Richard M. Ransohoff, Nobuhiko Ohno

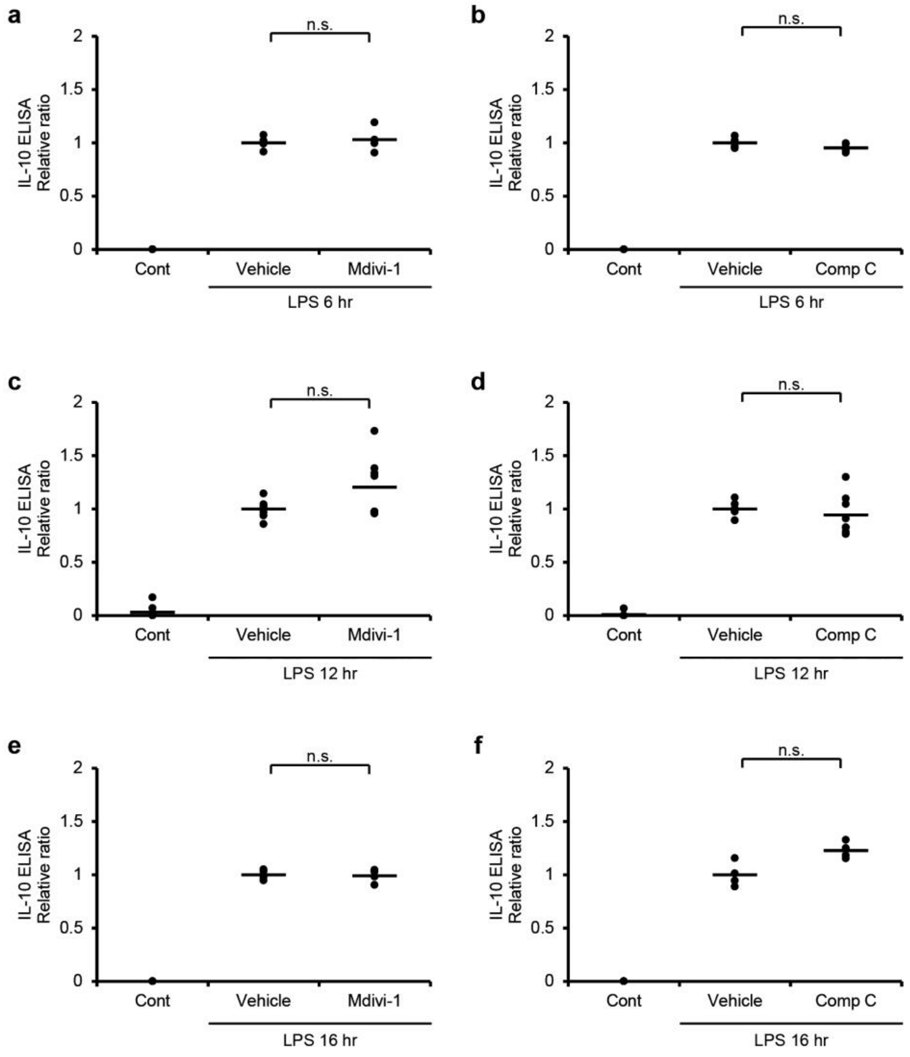

Supplementary Figure 1.

ELISA of IL-10 in supernatants of primary microglial cultures with or without (Cont) treatments of LPS for 6 hrs (a, b, LPS 6 hr), 12 hrs (c, d, LPS 12 hr), or 16 hrs (e, f, LPS 16 hr). Some of the cultures treated with LPS were also incubated with Mdivi-1 (a, c, e) or Compound C (b, d, f, Comp C). Each dot represents each replicate and means (bars) are shown. n.s.: not significant in t-tests.
